# Kinetic evaluation of a partially packed upflow anaerobic fixed film reactor treating low-strength synthetic rubber wastewater

**DOI:** 10.1016/j.heliyon.2020.e03594

**Published:** 2020-03-31

**Authors:** I. Nor Faekah, S. Fatihah, Zawawi Samba Mohamed

**Affiliations:** a Civil Engineering Department, Faculty of Engineering and Built Environment, Universiti Kebangsaan Malaysia, 43600 Bangi, Selangor, Malaysia; b Centre of Smart and Sustainable Township (SUTRA), Faculty of Engineering and Built Environment, Universiti Kebangsaan Malaysia, 43600 Bangi, Selangor, Malaysia

**Keywords:** Chemical engineering, Environmental chemical engineering, Biofuel, Water treatment, Environmental engineering, Waste treatment, Anaerobic digestion, Upflow anaerobic fixed film (UAF), Monod, Stover-Kincannon, Grau Second-Order

## Abstract

A bench-scale model of a partially packed upflow anaerobic fixed film (UAF) reactor was set up and operated at five different hydraulic retention times (HRTs) of (17, 14, 10, 8, and 5) days. The reactor was fed with synthetic rubber wastewater consisting of a chemical oxygen demand (COD) concentration of 6355–6735 mg/L. The results were analyzed using the Monod model, the Modified Stover-Kincannon models, and the Grau Second-Order Model. The Grau Second-Order model was found to best fit the experimental data. The biokinetic constant values, namely the growth yield coefficient (Y) and the endogenous coefficient (K_d_) were 0.027 g VSS/g COD and 0.1705 d^−1^, respectively. The half-saturation constant (K_s_) and maximum substrate utilization rate (K) returned values of 84.1 mg/L and 0.371 d^−1^, respectively, whereas the maximum specific growth rate of the microorganism (μ_max_) was 0.011 d^−1^. The constants, U_max_ and K_B,_ of the Stover-Kincannon model produced values of 6.57 g/L/d and 6.31 g/L/d, respectively. Meanwhile, the average second-order substrate removal rate, k_s(2)_, was 105 d^−1^. These models gave high correlation coefficients with the value of R^2^ = 80–99% and these indicated that these models can be used in designing UAF reactor consequently predicting the behaviour of the reactor.

## 1 Introduction

Anaerobic digestion was first introduced as a method for treating industrial and agricultural waste for decades. Anaerobic digestion has many advantages, the most important of which is that it can achieve both pollution control and energy recovery. The anaerobic digester must be designed to perform effectively so that it will not encounter any problems such as process instability or low methane yield.

Previous studies have improved upon the design of biological wastewater treatment reactors by mainly focusing on retaining the biomass within the reactor (Tay et al., 2006). A high-rate anaerobic reactor such as an upflow anaerobic filter (UAF) is one of the earlier designs with well-defined characteristics and operational parameters (Saravanan and Sreekri, 2006). At high loading rates, the continuous operation of packed up flow anaerobic filters may cause clogging to occur (Escudié et al., 2005). Therefore, low-density floating media were introduced as a novel solution to overcome this problem. This solution includes employing a kinetic model to model the design, operation, and optimization of a full-scale plant (Rajagopal et al., 2013).

A better understanding of the microbiology of an anaerobic digester and the process modifications, particularly fixed-film processes, has allowed anaerobic digesters to be used for dilute wastewaters and a large variety of industrial wastes. The development of the fixed-film filter is a significant achievement in anaerobic technology. The filter provides a relatively long solid retention time (SRT). Increased retention time makes it possible to treat moderate to low strength soluble organic industrial waste with a COD concentration of 2000–20,000 mg/L.

With the development of a mathematical model, the dynamic behavior of a process can be better understood. Furthermore, a kinetic model serves as a useful tool for understanding the underlying biological and transport mechanisms within a reactor (Acharyaa et al., 2011). Knowledge concerning the kinetic microbial growth rate, the substrate utilization rate, the limiting substrates or nutrients that affect the growth of cells, and the endogenous decay or death rate of microorganisms in the system is essential to ensuring the effective growth control and the proper balance of biomass in the system (Contreras et al., 2001).

The constants that are determined from the kinetic equation are called bio-kinetic coefficients or growth constants. These kinetic constants describe and predict the performance of the system. The bio-kinetic constants depend on the type of microbial species and the environmental conditions such as pH, temperature, dissolved oxygen, nutrients, inhibitory substances, and the degradability of the organic substrates in wastewater.

To date, kinetic modeling has been applied in a simplified form such that only a few parameters are involved to make the model easier to monitor and apply for industrial purposes and to determine the kinetic coefficients (Rajagopal et al., 2013). However, limited information is available on the process kinetics of substrate removal for low strength synthetic rubber waste water using upflow anaerobic fixed film reactor (UAF) reactor.

In this study, a partially packed upflow anaerobic fixed film reactor (UAF) was operated at different COD loading rates at ambient temperature conditions (28 °C–32 °C) in order to determine the kinetic constants involved in the process using kinetic models such as Monod model, the Stover-Kincannon models, and the Grau Second-Order model. The last part of this study is to compare the bio-kinetic coefficients with previous studies.

## 2 Materials and methods

### 2.1 Experimental setup

**Figure 1:**
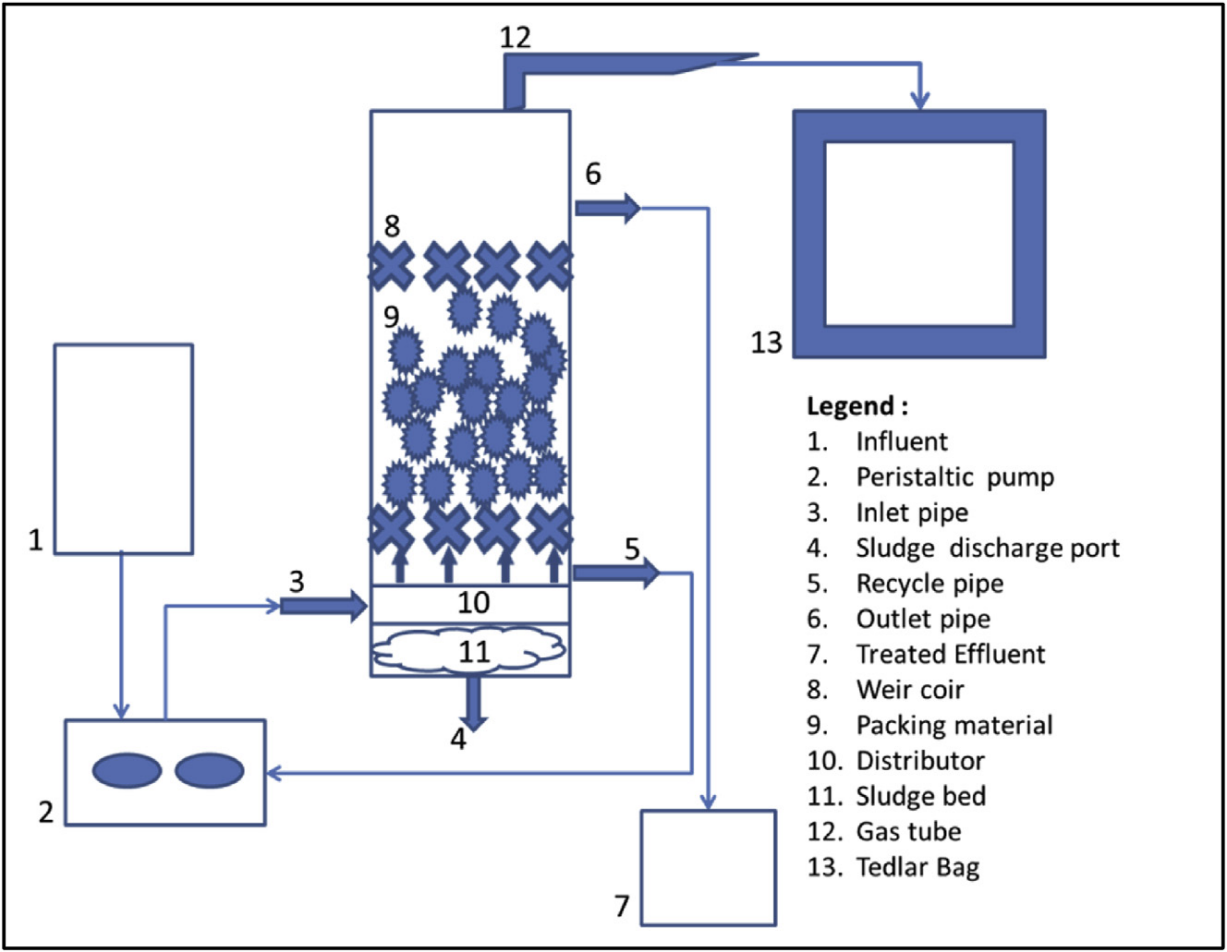
Schematic diagram of UAF reactor.

The UAF reactor used in this study is shown in Figure 1. The reactor consists of 5 main pipes: the feeding inlet pipe, the effluent outlet pipe, the recycle pipe, the gas pipe, and the sludge outlet pipe. Plexiglas having effective volume 7.0 L, internal diameter 15 cm, and effective height 50 cm was used in the study. All experiments were performed at ambient temperature (28 °C–32 °C) and no temperature control was imposed. A tubular polyvinylchloride (PVC) microbial filter 10 mm in height, 10 mm diameter and having density and specific surface area of 0.96 g/cm^3^ and 850 m^2^/m^3^ respectively. The UAF reactor was packed with 3116 pieces of media units, which equally about 40% of active volume of the reactor. These packing media were floated against a fixed screen (weir coil) at a height of 39.5 cm and placed 6.5 cm from the bottom of the anaerobic filters. To distribute the feed uniformly, an influent liquid distributor was mounted at the base of the column. Then, the substrate was continuously fed to the reactor through the base using a peristaltic pump (Cole Parmer, Masterflex L/S).

Biogas production was monitored daily until gas production can be negligible. A 3 L Tedlar Bag was used for daily collection of biogas through a valve mounted at the upper part of the digester. Displacement method was used to measure biogas production by measuring the downward displacement of water in the measuring cylinder and recorded difference of initial and final reading after feeding the digester. The reactor was fed from the bottom and the effluent was collected from the outlet provided at the top portion of the reactor.

### 2.2 Feed solution and digested sludge

The experiment was started by pumping about 0.5 L effluent at an initial loading rate of 0.1 g COD/l/d and a COD of 1.3 g/l daily. Then, the loading was gradually increased up to 0.4 g COD/l/d. The start-up of the reactor process took about 30 days to complete where the food-to-microorganism ratio and biomass content were monitored. After the reactor reached more than 80% COD removal rate, the operation to reduce the HRT commenced. The change to a different HRT was done once the reactor hydraulically reached to almost steady-state condition which was assumed to be reached when fairly constant biomass growth and permeate COD were attained. In order to determine this condition, it can be done by obtaining almost the same effluent COD concentration (standard deviation less than ±10%) for the last five consecutive operation days as considered by Kapdan and Erten (2007). The average value obtained from the bench-scale reactor under the effects of different hydraulic retention times and organic loading rate is presented in Table 1 whereas the feed solution characteristics was presented in Table 2. It was found that the wastewater has an average COD/N/P ratio of about 275/10/1. At this ratio, the wastewater was found to have sufficient amount of nutrients.

**Table 1:** Performance of model reactor (average value) during the experimental study.

HRT (days)	OLR (kg COD/m^3^.day)	COD_in_ (g/L)	COD removal efficiency,%
17	0.4	6.351	98.0
14	0.5	6.473	97.3
10	0.7	6.652	96.7
8	0.87	6	92.2
5	1.4	5.9	86.4

**Table 2:** Characteristics of the feed solution.

Parameter	Range
COD_soluble_	5900–6500
NH_3_–N	98–208
Total nitrogen	200–250
Total Phosphorus	70–90
Suspended Solids	-
Volatile Suspended Solids	-
pH	7.12

• All parameter units in mg/L except for pH.

A mixture of digested sludge obtained from an anaerobic pond of Malaysian Rubber Development Corporation (MARDEC) Berhad, Mentakab, Pahang was used for seeding. The digested sludge was used and contained 633,545 mg/L Total Solids (TS), 83,245 mg/L Volatile Solids (VS) and pH ranging from pH 6.62 to pH 6.92. Before 0.85 L of the mixture was loaded into the reactor, the mixture was passed through a screen to remove any debris. The reactor was left for 1 week to allow time for the sludge to stabilize.

### 2.3 Analytical procedure

The pH, chemical oxygen demand (COD), total suspended solids (TSS), volatile suspended solids (VSS), nitrogen, phosphorus, and alkalinity were analysed according to the methods described in Standard Methods for the Examination of Water and Wastewater (APHA, 1992). The COD was measured using a Hach DR 2010 spectrometer and a Hach COD reactor following the instructions provided for the Hach higher range test. The biogas composition was measured using a GA 5000 Geotech gas analyzer. All tests were performed in duplicate to obtain a consistent average. All analyses were undertaken at an ambient room temperature of 28 ± 2 °C.

### 2.4 Kinetic model application

The bio-kinetic coefficient was determined using a laboratory-scale study of the UAF reactor. The efficiency of the model reactor was evaluated based on its COD removal efficiency. In this study, the Monod, modified Stover-Kincannon, and Grau Second-Order models were applied using data obtained from the reactor operation.

#### 2.4.1 Monod model

In a biological treatment system, the rate of increase in biomass is directly proportional to the biomass concentration in the reactor. This proportionality factor is known as the specific growth rate constant (*U*). The formula is given below:

(1) $$\frac{1}{U}\;=\;\frac{\theta X}{\left(Si-Se\right)}\;=\;\frac{Ks}{K}\;.\;\frac{1}{Se}\;+\;\frac{1}{K}$$

where, ϴ, is hydraulic retention time (d); X, concentration biomass in the reactor (g VSS/L); $S_{i}$, influent substrate concentration (g/L); $S_{e,\;}$ effluent substrate concentration (g/L); $K_{S}$, half velocity constant (g/L) and *K,* maximum substrate utilization rate (d^−1^).

The yield coefficient, Y, is used to estimate the total amount of sludge produced as a result of wastewater treatment (Enitan and Adeyemo, 2014). The coefficient Y can be defined as the mass of new cells produced per unit of substrate utilized or removed by the microorganisms present in the treatment system. The equation as obtained below

(2) $$\frac{1}{\theta }\;=\;\frac{\left(\mathrm{Si}-\mathrm{Se}\right)}{\theta X}\;.\;Y-\mathrm{Kd}$$

where, ϴ, is hydraulic retention time (d); X, is the concentration biomass in the reactor (g VSS/L); $S_{i}$, is the influent substrate concentration (g/L); $S_{e,\;}$ is the effluent substrate concentration (g/L); Y, is the yield coefficient (gVSS/gCOD) and $K_{d}$ is the death rate constant (d^−1^).

The maximum specific growth of the bacteria, $\mu_{max}$ is related to the maximum specific substrate utilization rate. This growth occurs when the maximum substrate used is equal to the maximum rate of bacterial growth. The constant μ_max_ indicates maximum growth rate of microorganism when the substrate is being used at its maximum rate (Bhunia and Ghangrekar, 2008). Equation below shows the Michaelis-Menten equation that links the substrate removal with the specific growth rate of bacteria.

(3) $$\mu_{\text{max}}\;=K.Y$$

where, *μ*_max_ is the maximum specific growth rate of the bacteria (d^−1^); K is the maximum substrate utilization rate (d^−1^) and Y is the yield coefficient (gVSS/gCOD).

#### 2.4.2 Modified Stover-Kincannon Model

The Modified Stover-Kincannon model had been successfully applied in Rotating Biological contractor systems and biofilm reactors as per the study of Stover and Kincannon in 1982 (Stover and Kincannon, 1982). The special features of the modified Stover-Kincannon model are that the substrate utilization rate is expressed as a function of organic loading rate at steady state. The removal of the organic substrate in the anaerobic filter can be determined based on the substrate removal rate as a function of substrate concentration. Thus, at steady state, the form of the Stover-Kincannon model is presented by equation given below

(4) $$\frac{dS}{dt}=\;\frac{Q\left(Si-Se\right)}{V}=\;\frac{U_{\text{max}}\;\left(\frac{QSi}{V}\right)}{KB+\left(\frac{QSi}{V}\right)}$$

In linear form, equation above can be simplified to obtained as below equation

(5) $$\frac{dt}{dS}=\;\frac{V}{Q\left(Si-Se\right)}=\;\frac{KB}{U_{\text{max}}}\frac{V}{QSi}+\;\frac{1}{U_{\text{max}}}$$

Where, dS/dt is the substrate removal rate (g/L/d); Q, inflow rate (L/d); V, reactor volume (L); $S_{i}$, influent substrate concentration (g/L); $S_{e,\;}$ effluent substrate concentration (g/L); $U_{max}$, maximum utilization rate constant (g/L/d) and $K_{B}$, saturation value constant (g/L/d).

When written in terms of ϴ and its relationship with OLR, equation above becomes as given below

(6) $$\frac{\theta }{Si-Se}=\;\frac{KB}{U_{\text{max}}}\frac{1}{OLR}+\;\frac{1}{U_{\text{max}}}$$

where, ϴ, is hydraulic retention time (d); $S_{i}$, influent substrate concentration (g/L); $S_{e}$, effluent substrate concentration (g/L); $U_{max}$, maximum utilization rate constant (g/L/d) and $K_{B}$, saturation value constant (g/L/d).

By plotting the $\frac{V}{Q\left(Si-Se\right)}$, the inverse of the removal rate versus the $\frac{V}{QSi}$ , i.e. the inverse of the total loading rate, a straight line will be produced, with $\frac{1}{U_{\text{max}}}$ and $\frac{KB}{U_{\text{max}}}$ as the intercept and the slope of this line, respectively.

#### 2.4.3 Grau Second-Order model

(Grau et al., 1975) derived equation below as a general form of the second-order kinetic model;

(7) $$-\frac{dS}{dt}={k_{2}}_{\left(S\right)}X\;{\left(\frac{Se}{Si}\right)}^{2}$$

where, −dS/dt is the substrate removal rate (g/L/d); $k_{2\left(S\right)}$ is the second-order substrate removal rate constant (d^−1^); $S_{i}$, influent substrate concentration (g/L); $S_{e}$, effluent substrate concentration (g/L) and X, concentration biomass in the reactor (g VSS/L).

Equation above can be simplified and linearized to become as below

(8) $$\frac{Si\times HRT}{Si-Se}=HRT+\;\frac{\text{Si}}{K_{2}X}$$

(S_i_ − S_e_/S_i_) is expressed as the substrate removal efficiency (E) while the second term on the right-hand side is the constant, so equation above can be written as given below

(9) $$\frac{HRT}{E}=a+b*HRT$$

where $a=\;\frac{\mathrm{Si}}{{k_{2}}_{\left(S\right)}X}$ and b is a constant greater than unity. The kinetic constants ‘a’ and ‘b’ can be determined by plotting a graph of $\frac{\mathrm{HRT}}{E}\;$ versus HRT.

## 3 Results and discussion

The reactor was operated at five hydraulic retention times (HRTs) for about 350 days of operation. The feasibility results of UAF in treating synthetic rubber wastewater are presented with the organic loading rate varied from 0.5–1.3 g COD/L/day to assess the performance of the UAF reactor (Ismail and Suja, 2019). From the experimental results, the bio-kinetic coefficients obtained using the Monod, Stover-Kincannon and Grau Second-Order models were evaluated.

### 3.1 Kinetic analysis using the Monod model

**Figure 2:**
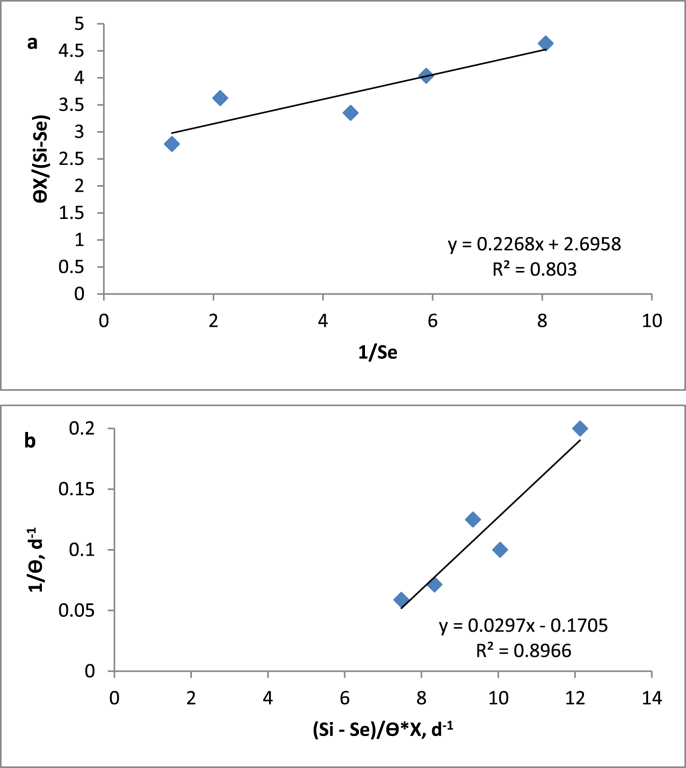
Monod model application to determine kinetic constants of a) Ks and K. b) Y and k_d_.

The Monod equation mathematically describes the relationship between the growth rate and substrate concentration using the maximum possible growth rate. Based on Eq. (1), the kinetic coefficients K_s_ and K can be determined from the experimental results by plotting a graph of $\frac{\theta X}{\left(\mathrm{Si}-\mathrm{Se}\right)}$ versus $\frac{1}{\mathrm{Se}}$. Figure 2a shows the straight line obtained from the curve-fitting method of the graphical data for the kinetic analysis.

According to (Bhunia and Ghangrekar, 2008), Eq. (2) can be used to estimate the k_d_ and Y values by plotting a linear regression of $\frac{1}{\theta }$ against $\frac{\mathrm{Si}-\mathrm{Se}}{\theta X}$ .The intercept from this line is equal to k_d_ whereas Y is the slope of the straight line that passes through the plotted points as shown in Figure 2b.

Using this model, the bio-kinetic coefficients obtained are as below:

The maximum substrate utilization rate constant, K = 0.371 d^−1^; the half unloading or saturation constant, K_s_ = 0.0841 g/L; the endogenous decay coefficient, k_d_ = 0.1705 d^−1^; the yield coefficient, Y = 0.0297 mg VSS/mg COD; and the maximum specific growth rate of bacteria, μ_max_ = 0.011 d^−1^. From this model application, coefficient of determination obtained was quite high as R^2^ = 0.8–0.9.

The value of K_s_ as estimated by the model (84.1 mg/L) was far from the K value (0.371 d^−1^). This condition is favorable, as the process efficiency will not be reduced when OLR increases, as pointed out by Ahn and Foster (2000). Previous studies proposed that the higher K_s_ value will results the higher biodegradability of substrates (Ahmadi et al., 2015). The value of K is an indicator of the ability of microorganisms to degrade the substrate present in the waste and to produce methane (Enitan and Adeyemo, 2014). A high K value indicates that it is significantly difficult to convert organic matter to methane inside the reactor (Fdez-Güelfo et al., 2012). In addition, from the K value, biomass concentration in UAF can be estimated because it is very difficult to calculate the biomass concentration on support media in anaerobic reactor (Bhunia and Ghangrekar, 2008).

Meanwhile, a large K_d_ value was obtained from the graph, indicating that the net sludge volume produced or to be handled was high.

### 3.2 Kinetic analysis using the Stover-Kincannon Model

The Stover-Kincannon model expresses the substrate utilization rate as a function of organic loading rate in a biofilm reactor (Sentürk et al., 2010). In the modified version of this model, the volume of the reactor is used instead of the surface reactor volume (Ahn and Foster, 2000). This model gives a high correlation compared to other models and has been widely used to determine the biokinetic coefficients of a contact growth system.

**Figure 3:**
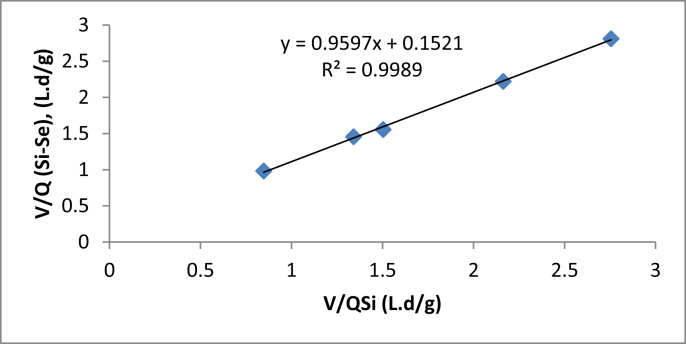
Determination of kinetic constants Umax and KB using Stover-Kincannon Model.

By using the data in Table 3, a graph was plotted, as shown in Figure 3. The plot of experimental data was based on the linearized equation at steady state as in Eq. (4), where a high correlation (R^2^ = 0.9989) was obtained. $\frac{1}{Umax}$ and $\frac{KB}{Umax}$ were calculated as 0.1521 and 0.9597, respectively. The maximum removal rate constant (U_max_) was 6.57 g/L/d and the saturation value constant (K_B_) was 6.31 g/L/d.

**Table 3:** Data used to determine U_max_ and K_max_.

Q (L/d)	S_i_ (g/L)	S_e_ (g/L)	V/QS_i_ (L d/g COD)	V/[Q (S_i_– S_e_)] (L d/g COD)
0.4	6.351	0.124	2.755	2.810
0.5	6.473	0.17	2.163	2.221
0.7	6.652	0.222	1.503	1.555
0.87	6	0.471	1.341	1.455
1.4	5.9	0.805	0.847	0.981

The value of K_B_ was low, indicating that the UAF has a low potential in coping with high-strength wastewater (Sentürk et al., 2010). The close values between U_max_ and K_B_ indicate that the process efficiency will decrease as organic loading rate increases, as reported by Ahn and Foster (2000).

By substituting the value of K_B_ and U_max_ into equation below, the effluent COD concentration, S_e_, can be predicted using the below equation.

(10) $$Se=Si-\frac{U_{\text{max}}\;.Si}{KB+\left(\frac{QSi}{V}\right)}$$

### 3.3 Kinetic analysis using the Grau Second-Order kinetic model

**Figure 4:**
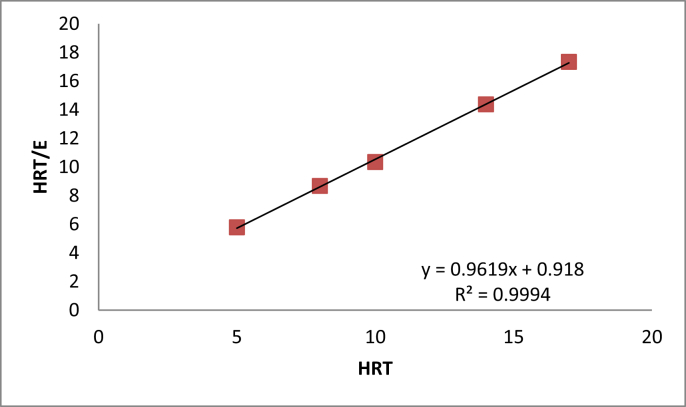
Second-order model application.

By plotting $\frac{HRT}{E}$ versus HRT as shown in Figure 4, a straight line with an R^2^ value of 0.9994 is produced. The reciprocal and slope of the line represent the kinetic constant ‘b’ and ‘a’ with values of 0.918 and 0.9619, respectively. The second-order substrate removal rate constant, k_2(s)_ in the unit of time, was derived from the linear equation of Eq. (8), which was calculated from $\;a=\;\frac{Si}{k_{2(S)}X}$ for UAF and listed in Table 4.

**Table 4:** Data for the second-order kinetic model.

HRT,d	S_i_, g COD/L	S_e_, g COD/L	X, g VSS/L	E (%)	HRT/E	k_2(S),_d^−1^
17	6.351	0.124	0.049	98.0	17.34	135
14	6.473	0.17	0.054	97.3	14.38	125
10	6.652	0.222	0.064	96.7	10.35	108
8	6	0.471	0.074	92.2	8.681	84.3
5	5.9	0.805	0.084	86.4	5.790	73.0
Average						105

The COD concentration of the effluent substrate can be predicted by rearranging Eq. (9) to become as below equation.

(11) $$S=Si\left(1-\;\frac{HRT}{a+b*HRT}\right)$$

### 3.4 Evaluation of kinetic models in UAF reactor

Table 5 summarizes the substrate removal kinetic constants cited in the literature based on the different types of reactors and wastewater used. Many researchers have arrived at different values of kinetic constants using various substrates and reactors.

**Table 5:** Summary of kinetic constants obtained cited in the literature with the present study.

Name of Model	Reactor Type	Substrate/waste water	Influent COD concentration (mg/L)	HRT (days)	Obtained kinetic constants values				References
					Y, g/g	k_d_,d^−1^	K_s_, mg COD/L	K, d^−1^	
Monod	AH	Petrochemical Waste	1000–4000	0.17–2	0.132	0.121	1116	0.487	(Jafarzadeh Mehrdadi and Hashemian, 2009)
	UASB	Brewery waste water	1000–3000	0.3–6.5	0.882	0.083	0.046	-	(Enitan and Adeyemo, 2014)
	CSTR	Volatile fatty acid mixture	-	-	0.03	0.099	-	17	(Alphenaar, 1994)
	ABR	Dairy waste water	20,000–34,000	10.5–20	0.24	0.06	1310	0.20	(Shoba, 2009)
	UASB	Synthetic waste water	300–400 mg/L	0.13–0.33	0.083	0.006	226.1	0.699	(Bhunia and Ghangrekar, 2008).
	AnFFBR UAnFFBR	Diethyl phthalate	300–700	0.5–1.5	0.156–0.146	0.107–0.1	31.34 24.87	1.13 1.03	(2017)
	UAF	Synthetic rubber waste water	6355–6375	5–17	0.0297	0.1705	84.1	0.371	Present study
					U_max_, g/L/d			K_B_, g/L/d	

Stover Kincannon	UAF	Simulated fruit canning waste water	9000–11600	0.5	109.9			109.7	(Rajagopal et al., 2013)
	UAF	Cheese dairy waste water	23000–40000	1.6	53.5			49.7	(Rajagopal et al., 2013)
	UAFB	Textile waste water	1800–3800	0.4–1	31.69			45.37	(Sandhya and Swaminathan, 2006)
	Mesophilic AF	Simulated starch	-	0.25–1	49.8			50.6	(Ahn and Foster, 2000)
	MACR	Potato processing waste water	5200–5700	1.06–5.11	22.93			23.59	(Sentürk et al., 2010)
	UAFB	Formaldehyde containing waste water	10 976–11 840	0.4–1	3.4			4.6	(Raja Priya et al., 2009)
	UAFB	Synthetic rubber waste water	6355–6375	5–17	6.57			6.31	Present study

					k_2(s)_, d^−1^			ab	

Second Order	UAFB	Formaldehyde containing waste water	10 976–11 840	0.4–1	3.2 h^-1^		0.64	9.36	(Raja Priya et al., 2009)
	UAF	Simulated fruit canning waste water	9000–11600	0.5	5.0		0.08	1.0	(Rajagopal et al., 2013)
	UAF	Cheese dairy waste water	23000–40000	1.6	1.93		0.56	0.92	(Rajagopal et al., 2013)
	UAFB	Textile waste water	1800–3800	0.4–1	10.50 h^-1^		0.9151	5.1386	(Sandhya and Swaminathan, 2006)
	UAFB	Synthetic rubber waste water	6355–6375	5–17	105		0.918	0.962	Present study

∗ AH - Anaerobic hybrid, UASB – Upflow sludge blanket, ABR – Anaerobic baffle reactor, CSTR – Continuous stirred tank reactor, UAF – Upflow anaerobic filter, UAFB – Upflow anaerobic fixed film, MACR Mesophilic anaerobic contact reactor.

Based on the Monod model, the value of Y and μ_max_ determined from this study were 0.0297 mgVSS/mgCOD and 0.011 d^−1^ respectively was quite near with the study conducted by Bhunia and Ghangrekar (2008) for synthetic waste water having COD concentration in the range of 300–400 mg/L in UASB reactor with the Y and μ_max_ value as 0.083 mgVSS/mgCOD and 0.058 d^−1^ respectively. Alphenaar (1994) also determined the same value for kinetic coefficients of Y as 0.03 mgVSS/mgCOD for volatile fatty acid mixture but larger value for μ_max_ (0.51 d^−1^). Yousefzadeh et al. (2017) reported higher value of $\mu_{max}$ as 0.176–0.151 d^−1^ for diethyl phthalate removal using anaerobic fixed film baffled reactor (AnFFBR) and up flow anaerobic fixed film fixed bed reactor (UAnFFFBR). Meanwhile, larger value of k_d_ (0.1705 d-1) obtained in this study compared to Bhunia and Ghangrekar (2008) f as the value was 0.006 d^−1^whereas K_s_ value of 84.1 mg/L has different values from other researcher.

When applying the Stover-Kincannon model, the value of the kinetic constant was found similar to that of (Raja Priya et al., 2009). The U_max_ and K_B_ values reported for formaldehyde containing waste water in UASB were slightly lower, at 3.4 g/L d^−1^ and 4.6 g/L d^−1^, respectively whereas larger value was obtained in this study using UAF which was the value of $U_{max}$ and $K_{B}$ were 6.57 g/L/d and 6.31 g/L/d respectively. Meanwhile, the findings of the rest of the studies reported have far value compared to the results obtained in the present study. For instance, Rajagopal et al. (2013) reported U_max_ and K_B_ values of 109.9 g/L/d and 109.7 g/L/d respectively for fruit canning waste water and 53.5 g/L/d and 49.7 g/L/d respectively for cheese dairy waste water using UAF which was packed with low-density polyethylene media filled about 80% of active volume of the reactor. Higher value of $U_{max}$ and $K_{B}$ demonstrated that microbial community achieved good biodegradable conditions of substrates and consequently stabilizing COD in the reactor (Yousefzadeh et al., 2017).

Similarly, the kinetic constants obtained using the Grau second-order model was also found to be different and far compared to other kinetic studies as stated in Table 5. Rajagopal et al. (2013) agrees with this statement, and conclude regardless of any substrate concentration, substrate removal rates were mainly depends on the nature of the substrate, the microorganism living in the reactor and reactor configuration. Kinetic parameters for high rate reactors such as fixed bed reactors are apparent values as they embody all the mass transfer parameters. As shown in Table 4, the values of substrate removal rate constant, $k_{2\left(S\right)}$ obviously decreased as HRT decreased even when the microbial community in the reactor increased.

In conclusion, the kinetic coefficients obtained in these study provides good agreement with all the models applied. Thus, the result of kinetic studies obtained from lab-scale experiments can be used in the design of UAF with partially packed media and also for estimating treatment efficiency of full-scale reactors with low to medium strength waste water applied.

## 4 Conclusion

The performance of UAF in treating synthetic rubber processing wastewater with a COD concentration of 5900–6600 mg/L was evaluated at different HRTs and OLRs. All kinetic models were found capable of describing the bio-kinetic behavior in the UAF reactor with good correlation. The kinetic coefficients derived from this waste water treatment using UAF with half partially packed with PVC as support media provides good agreement with the Stover-Kincannon and Grau second-order models. In the future research, one has to ensure that the selection for good inoculum is vital. This is because the optimum inoculum to substrate ratio depends on the source of inoculum. The different sources of inoculums will have different metabolic activities so the optimum ratio required for optimum anaerobic digestion of a particular feed may vary using inoculums from different sources. Therefore, it is proposed to have the same inoculum so that it can meet similar results.

## Declarations

### Author contribution statement

NOR FAEKAH I.: Performed the experiments; Analyzed and interpreted the data; Wrote the paper.

FATIHAH S.: Conceived and designed the experiments; Contributed reagents, materials, analysis tools or data.

### Funding statement

This research has received funding from the Ministry of Higher Education Malaysia FRGS/1/2013/TK07/UKM/02/05.

### Competing interest statement

The authors declare no conflict of interest.

### Additional information

No additional information is available for this paper.
